# Valorization of Spent Grains from Beer Production through β-Glucan Extraction

**DOI:** 10.3390/foods13030440

**Published:** 2024-01-29

**Authors:** Natcha Jantason, Manop Suphantharika, Angkana Wipatanawin, Suwan Chansong, Panwajee Payongsri

**Affiliations:** 1 School of Bioinnovation and Bio-Based Product Intelligence, Faculty of Science, Mahidol University, Rama 6 Road, Bangkok 10400, Thailand; natchajajah@gmail.com (N.J.); manop.sup@mahidol.ac.th (M.S.); angkana.wip@mahidol.ac.th (A.W.); 2 Department of Biotechnology, Faculty of Science, Mahidol University, Rama 6 Road, Bangkok 10400, Thailand; 3 Singha Beverage Co., Ltd. (Branch No. 00001) 99 Moo 10, Buapaktha, Nakorn Pathom 73130, Thailand; suwan_c@boonrawd.co.th

**Keywords:** β-glucan, brewers’ spent grain, functional properties, hydrocolloid, beverage

## Abstract

Brewers’ spent grains (BSG) are the major byproduct of the brewing industry. Recently, it has been found that β-glucan, which can be used as a food supplement, can be extracted from BSG and offers the greatest added value. This study aimed to investigate the effects of temperature (45–90 °C) and time (30–120 min) on β-glucan extraction efficiency when using hot water extraction. β-glucan was precipitated upon 80% ethanol addition. The chemical compositions were examined. The highest β-glucan concentration and yield were obtained at a temperature and time of 60 °C and 90 min, respectively. The functional properties of the extracted β-glucan were analyzed and compared with other commercial stabilizers such as sodium carboxymethyl cellulose (CMC), xanthan gum, gum arabic, and oat β-glucan. All stabilizers exhibited non-Newtonian flow behavior, except for gum arabic, which exhibited Newtonian flow behavior. The water holding capacity of BSG β-glucan was 6.82 g/g and the creaming index of the emulsions stabilized with BSG β-glucan was 89.05%. BSG β-glucan improved the color and stability of orange juice by reducing the precipitation of orange pulp. This study illustrated that BSG β-glucan can be used as a stabilizer and viscosity enhancer in foods, depending on the concentration, which can be applied to a variety of foods.

## 1. Introduction

The brewing industry produces large amounts of waste and byproducts, including spent grains, spent yeast, and spent hops. Spent grains are the most common byproduct in the brewing process [1]. The most common usage of BSG has been in animal feed production, which has a relatively low value. Much research has focused on BSG valorization processes to generate products suitable for various applications. These include human nutrients, energy production, hydroxycinnamic acid synthesis, xylitol, and brick components [2]. Dietary fibers are also one of the products that have gained interest in the brewing industry.

Barley contains 2–5% soluble dietary fiber and 70% of the total dietary fiber is β-glucan [3]. These non-starchy polysaccharides consist of mixed (1→3) and (1→4) β-linkages. β-glucan from brewers’ spent grains (BSG) can be used as a hydrocolloid [4]. Barley β-glucan shows great potential as a thickener or stabilizer in food products [5]. β-glucan also has a positive effect on health by lowering cholesterol levels in blood serum and regulating blood glucose levels [6]. These, together with the large quantity of spent grains from the brewing industry, have attracted the brewing industry to recovering β-glucan from BSG to be used as a value-added product.

β-glucan from cereal is soluble in hot water and alkaline solutions, while it precipitates in ammonium sulfate and ethanol. Therefore, the hot water extraction process is usually applied in β-glucan extraction. An enzymatic step may be applied to further remove starch and protein [7]. As illustrated in the study of Ahmad et al. [7], the highest yield, water binding, and foaming capacity of barley β-glucan was found with the hot water extraction method. BSG differs from barley in that it is germinated during malting and mashed during the brewing process. The BSG β-glucan has a lower average molecular mass than the barley β-glucan, which is most likely due to the degradation of the β-glucan during malting and mashing [8]. Currently, there are some reports in the literature on the extraction and characterization of β-glucan from BSG [4,8,9], but there are no reports on the potential applications of the β-glucan from BSG as a functional ingredient in the real food system. In the extraction of β-glucan from BSG by hydrothermal treatment, the treatment temperature and reaction time have an important effect on the extraction and properties of β-glucan [4]. The physical, chemical, and functional properties of β-glucan can be influenced by the extraction conditions [10]. Studying the functional properties of β-glucan extract from BSG allows us to determine the amount and concentration to be used in the appropriate product. To ensure that the β-glucan extracted from BSG is comparable to the commercial product, these properties must be studied.

Therefore, the aim of this study was to investigate the effects of temperature and time on the extraction of β-glucan from BSG, to compare the functional properties of BSG β-glucan with other commercially available stabilizers, and finally to use BSG β-glucan in beverage products.

## 2. Materials and Methods

### 2.1. Materials

Brewers’ spent grain (BSG) was supplied by a commercial brewery in Nakorn Pathom, Thailand. It was a mash mixed with barley malt and rice, which is produced during the brewing of lager beer. Gum arabic (CAS No.: 9000-01-5) and sodium carboxymethyl cellulose (CMC) (CAS No.: 9004-32-4) were purchased from Krungthepchemi Co, Ltd., Ladprao, Bangkok, Thailand. Xanthan gum (CAS No.: 11138-66-2) was purchased from JR F&B Co., Ltd., Lat Krabang, Bangkok, Thailand. Their specifications provided information on the viscosity at a 1% solution with the measured viscosities as follows: CMC, 1800–2000 mPa·s; xanthan gum, 1200–1600 mPa·s; gum arabic, 60–110 mPa·s. Oat β-glucan was purchased from Shaanxi Rebecca Bio-Tech Co., Ltd., Xi’an, Shaanxi, China. All specifications are provided in the Supplementary Materials. The main components of the BSG were analyzed by standard analytical methods [11,12]. The carbohydrate content of the BSG was 64.60% (AOAC Method 986.25), the insoluble dietary fiber content was 57.96% (AOAC Method 991.43), the soluble dietary fiber content was 2.39% (AOAC Method 991.43), and the ash and moisture contents were 3.27% (AOAC Method 920.153) and 6.15% (AOAC Method 950.46 (B)), respectively. The β-glucan content of the BSG analyzed with a β-glucan assay kit (Megazyme Pty. Ltd., Wicklow, Ireland) was 0.455% (dry weight). The starch content in the BSG was 0.7% as determined by Megazyme kit, K-TSTA-50A (AOAC Method 996.11). Canola oil was purchased from a local supermarket. All reagents were of analytical grade.

### 2.2. Extraction of β-glucan from BSG

The extraction of β-glucan was performed according to the hot water method described in [7] and [13] with some modifications. One hundred grams of BSG were suspended in 700 mL of distilled water. The mixture was then heated at different temperatures (45, 50, 55, and 60 °C) and times (30, 60, 90, and 120 min). A thermostable alpha-amylase (Thermamyl 120 L; Novozyme, Bagsvaerd, Denmark) 0.6% (*v*/*v*) was added to the solution to hydrolyze the remaining starch (at 90 °C, 1 h) [13]. The mixture was centrifuged for 10 min at 3000× *g* (NF400 Santrifuj; Ankara, Turkey) to remove the insoluble residue. The pH was then adjusted to 4.5 with 2 M acetic acid and then heated at 95 °C in a water bath before separating the precipitated proteins by centrifugation at 3000× *g* for 10 min [14]. The supernatant was adjusted to pH 7 with 2M NaOH. The β-glucan was precipitated by adding ethanol (80%) to the supernatant with stirring in a 1:1 ratio and stored overnight at 4 °C; the solution was then centrifuged at 4 °C at 4500× *g* and the sample was dried overnight up to constant weight at 40 °C [7]. All the material obtained after extraction, containing β-glucan and other impurities, is referred to here as extract. The extraction yield of β-glucan is the weight of β-glucan in grams extracted from 100 g of BSG. The recovery yield of β-glucan is the extraction yield of β-glucan divided by the β-glucan content of the BSG multiplied by 100.

### 2.3. Functional Properties Analysis of the Extract

#### 2.3.1. Water Holding Capacity (WHC)

The water holding capacity (WHC) of the samples was measured according to the modified method described by [7]. Twenty milliliters of distilled water was placed in a centrifuge tube containing 200 mg of β-glucan, and other samples were prepared by weighing the sample to 1 g and adding 10 g of distilled water. The tubes were mixed with a vortexer for 10 s every 5 min for 30 min and centrifuged at 1000× *g* for 15 min. The supernatant was discarded and the weights of the tube and pellets were recorded. The WHC was calculated using the following equation:

WHC = (wet sample weight − dry sample weight)/dry sample weight(1)

#### 2.3.2. Viscosity

For viscosity measurement, 0.5% (*w*/*v*) dispersions of β-glucan and commercial stabilizers in water were prepared by heating in a boiling water bath for 5 min followed by continuous stirring for 2 h at room temperature. The viscosity of the dispersions was determined using a Brookfield RVDV-III viscometer (Brookfield Engineering Laboratories, Inc., Stoughton, MA, USA) with UL adapter spindle UL00 at 25 °C and shear rates of 1.22–97.8 s^−1^. A concentration of 0.5% was chosen to ensure a comparison of all stabilizers within the range of measurable viscosity—A suitable range for measurement and comparison.

#### 2.3.3. Emulsion Stabilizing Capacity

The aqueous solution was prepared using 0.5% (*w*/*v*) of various stabilizers and dissolved in deionized water. It was then stirred at 10,000 rpm for 15 min in an Ultra Turrax T18 homogenizer (IKA Works, Inc., Wilmington, NC, USA). The solution was then heated to 80 °C for 10 min with constant stirring, cooled to room temperature, and the pH was adjusted to 4. Before emulsion formation, the solutions were left overnight at refrigerator temperature (4 °C). Emulsions were prepared by mixing 20% (*v*/*v*) canola oil and 80% (*v*/*v*) aqueous phase and homogenized at 10,000 rpm for 5 min at room temperature and used for creaming index and zeta potential measurements.

A creaming index to evaluate the relative stability of O/W emulsions was measured. The freshly prepared emulsions (10 mL) were filled into glass test tubes with a diameter of 20 mm and a height of 60 mm and then sealed to avoid evaporation. The sample tubes were stored at room temperature for 7 days and creaming boundaries were seen moving over time. Several emulsions separated into an upper cream layer and a lower serum layer during storage. The height of the serum layer (H_S_) and the total height of the emulsions (H_E_) were measured on day 1 and day 7 of storage. The creaming index (CI) is defined by the following equation [12]:

CI (%) = 100 × (H_S_/H_E_) (2)

The zeta potential of the stabilized O/W emulsions with different stabilizers was analyzed using a particle electrophoresis instrument (Zetasizer Nano ZS90, Malvern Instruments Ltd., Worcestershire, UK). Prior to testing, samples were diluted with distilled water to a concentration of 0.005% (*w*/*w*). Three readings were taken from each of the three freshly prepared samples. The zeta potential was expressed as the mean and standard deviation of these data.

The protein content of the extracted BSG β-glucan was determined by the Bradford assay using bovine serum albumin (BSA) as a standard. One hundred microliters of BSA (0.1 mg/mL) or β-glucan (10 mg/mL) solutions in distilled water were mixed with 1 mL of Bradford reagent, incubated for 5 min at room temperature, and absorbance was measured at 595 nm. A standard curve was constructed, and the protein concentration of the β-glucan solution was determined by comparing the measured values with the curve of the BSA standard solution.

### 2.4. Beverage Preparation and Characterization

Sai Nam Phueng oranges (Citrus reticulata Blanco) were purchased in the local markets of Bangkok, Thailand. Fresh orange juice was prepared using an electric citrus juicer (model JE-341A, Otto King Glass Co. Ltd., Bangkok, Thailand). The oranges were cut in half with a sharp knife and each half was pressed onto the spinning reamer and the orange half was turned until the juice was squeezed out. The extracted juice was filtered through a muslin cloth and divided into different portions to stabilize with 1% (*w*/*v*) β-glucan [15], 0.2% CMC, 0.1% xanthan gum, and 1% gum arabic. The orange juice prepared without stabilizer served as a control. The mixture was mixed for 15 s, heated to 75 °C in a water bath for 15 min and then cooled to room temperature. The apparent viscosity of the product prototype was measured at shear rates of 1.22–97.8 s^−1^ using a Brookfield RVDV-III viscometer with UL adapter spindle UL00, and its color was measured using a Hunterlab ColorFlex EZ spectrophotometer (Maha Chemicals Asia, Pte. Ltd., Singapore). A Zetasizer Nano ZS90 was used to measure the zeta potential.

### 2.5. Statistical Analysis

All measurements were performed in triplicate for each sample. Results are reported as calculated means and standard deviations. Statistical analyses were performed using SPSS version 25.0 (SPSS Inc., Chicago, IL, USA) to determine the significant difference between means (*p* ≤ 0.05) using a one-way ANOVA test.

## 3. Results and Discussion

### 3.1. Effect of Temperature and Time on β-Glucan Extraction

β-glucan was extracted from BSG by hot water extraction at different temperatures (45, 60, 75, 90 °C) and times (30, 60, 90, 120 min). The average concentrations of β-glucan in the extract obtained at different temperatures and times are shown in Table 1. The highest purity of the β-glucan in the extract, at 59.84% (*w*/*w*), was obtained at a temperature of 60 °C and an extraction time of 90 min. The β-glucan recovery should be considered when evaluating the extraction efficiency under different conditions. The β-glucan recovery yield was determined from the weight of β-glucan in the extract divided by the weight of β-glucan in the raw material. The percentage of β-glucan recovered at 60 °C and 90 min, of 12.86% (*w*/*w*), was the highest among the other conditions. The yield of the extract does not represent the total amount of β-glucan. In addition to β-glucan, this extract also contained some protein, ash, and starch. The highest extraction yield of β-glucan, of 0.057% (*w*/*w*), was obtained at 60 °C and 90 min. This yield corresponds to the weight of β-glucan in grams extracted from 100 g of BSG. The native state of the extracted hemicellulose can be obtained at low temperatures (100 °C), but it is not possible to break the cross-links between the lignocellulose components, resulting in a low extraction yield and a long extraction time [16,17].

A similar method of hot water extraction was used by Zielke et al. [8] at 100 °C and 90 min, where only 1.4 g β-glucan/100 g extract was obtained, which is significantly lower than the 59.8 g β-glucan/100 g extract obtained in this study at an optimal condition of 60 °C and 90 min. This difference could be attributed to the different method of purification. The β-glucan in this study was purified by precipitation with ethanol, in contrast to dialysis in [8], which cannot remove large molecules such as some hydrolysis products of starch. However, at the higher product purity obtained in this study, the extraction yield (0.057%, *w*/*w*) was significantly lower than that reported in [8] (13%, *w*/*w*). A much higher extraction yield and recovery yield of β-glucan of 0.63% (*w*/*w*) and 85.1% (*w*/*w*), respectively were obtained by hydrothermal treatment of BSG at 160 °C, 2 min, and a pressure of 20 bar [4]. However, most of the extracted β-glucan (more than 80%) was degraded to smaller molecules with a molecular weight of less than 50 kDa, which is undesirable as it has less health-promoting effect [18]. In addition, our study showed that the extraction of β-glucan using hot water resulted in higher β-glucan purity (59.8%, *w*/*w*) compared to the hydrothermal extraction (0.63%, *w*/*w*). The low product purity could be due to the fact that the β-glucan was not purified from the BSG hydrolysates [4]. Steiner et al. [4] illustrated that the arabinoxylan in the extract from hydrothermal treatment of BSG was nearly 30 times higher than that of β-glucan. Consequently, the extract from hydrothermal treatment would be expected to contain less than 3 g β-glucan/100 g extract. Moreover, undesirable heat-induced degradation products, namely 5-hydroxymethylfurfural (HMF) and furfural, also occurred in the BSG during hydrothermal treatment, which could pose a health risk [4]. Therefore, the hot water extraction performed in this study at milder extraction conditions is of great interest to minimize the degradation of β-glucan and the formation of undesirable degradation products. In addition, this extraction was carried out at ambient conditions so that no high-pressure vessel is required, which is not only more cost-effective but also requires lower safety standards.

With higher β-glucan content, the application of the extract can be extended for purposes such as fortifying milk to reduce calories as shown by Sharafbafi et al. [19]. β-glucan has also been incorporated into beverages to reduce energy intake, as indicated by [20]. It is noteworthy that the FDA has granted Generally Recognized as Safe (GRAS) status to β-glucan from barley extracted through hot water treatment [21]. This regulatory approval allows the brewing industry to directly adopt this extraction method.

To determine the optimal conditions for the factors influencing the extraction of β-glucan from BSG, i.e., extraction temperature and time, the classical variation approach was used in this study and in the study by Steiner et al. [4]. However, the response surface methodology will be used for optimization in future work as it has several advantages over the classical variation approach, i.e., smaller number of experiments, determination of the absolute maximum, and identification of possible interactions between the independent parameters.

### 3.2. Comparison of the Functional Properties of BSG β-glucan with Other Commercial Stabilizers

The functional properties of BSG β-glucan were compared with those of β-glucan from oats, carboxymethyl cellulose (CMC), gum arabic, and xanthan gum. β-glucan from BSG and commercial stabilizers were investigated for viscosity, emulsion stabilizing capacity, and water holding capacity.

#### 3.2.1. Apparent Viscosity

**Figure 1:**
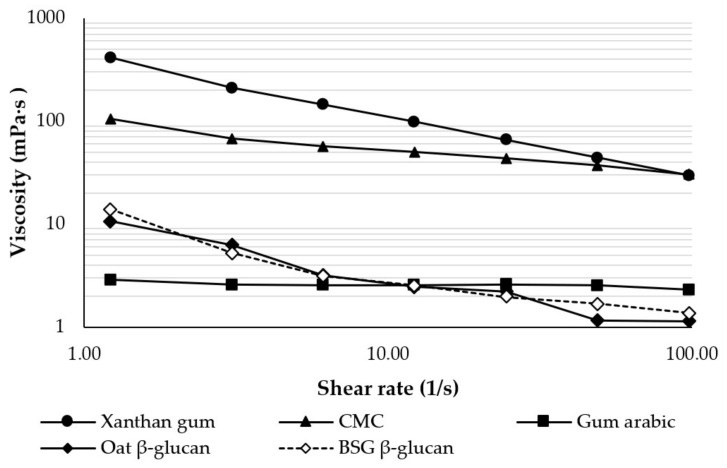
Apparent viscosity as a function of shear rate of various stabilizers at a concentration of 0.5% (*w*/*v*) and 25 °C. CMC = sodium carboxymethyl cellulose, BSG = brewers’ spent grain.

The apparent viscosity of the different stabilizers at the same concentration and a shear rate range of 1.22–97.8 s^−1^ is shown in Figure 1. Xanthan gum had the highest viscosity, followed by CMC, BSG β-glucan, oat β-glucan, and gum arabic. All stabilizers, except gum arabic, showed a decrease in apparent viscosity with increasing shear rate, indicating their non-Newtonian, shear-thinning (pseudoplastic) flow characteristics. This could be due to the breaking of hydrogen bonds in the samples, caused by strong mechanical shearing at higher shear rates [22]. According to a study on β-glucan from barley [23], β-glucan can act as a viscosity improver, and viscosity increases with the concentration and purity of β-glucan. Gum arabic, on the other hand, exhibited a relatively constant apparent viscosity over the shear rate range studied, indicating its Newtonian flow characteristics, which is consistent with an earlier study [24].

#### 3.2.2. Water Holding Capacity (WHC)

**Figure 2:**
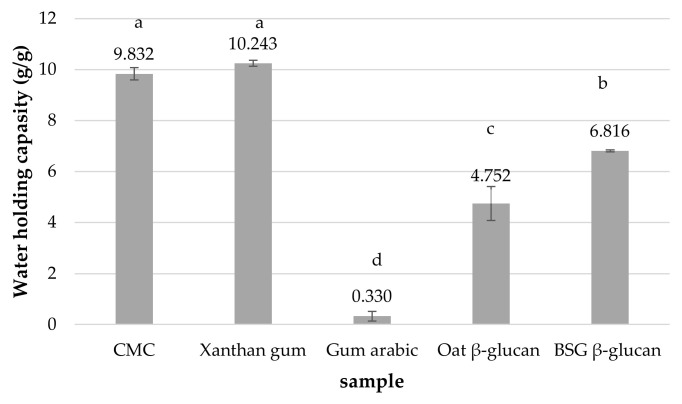
Water holding capacity of the different stabilizers. Different letters indicate significant differences (*p* < 0.05 by Duncan’s multiple range test). CMC = sodium carboxymethyl cellulose, BSG = brewers’ spent grain.

The water holding capacity (WHC) of the different stabilizers decreased in the following order: xanthan gum ≈ CMC > BSG β-glucan > oat β-glucan > gum arabic, as shown in Figure 2. Shrinkage of the gel did not occur at high WHC levels. This prevents some liquid from leaking out of the gel. It can avoid syneresis in food. Compared to oat β-glucan, BSG β-glucan has a higher WHC value, which affects the shelf life of the product [7].

According to Ahmad et al. [7], the highest value of WHC (3.79 g/g DW) was found in barley β-glucan extracted by hot water treatment, suggesting that this material could be successfully used as a functional ingredient to prevent syneresis in various foods such as jams, jellies, and sauces.

#### 3.2.3. Emulsion Stabilizing Capacity

**Figure 3:**
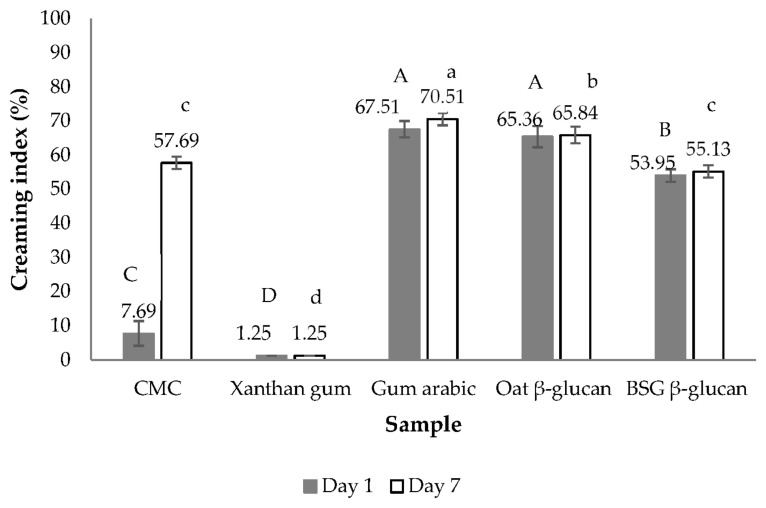
Creaming index of emulsions stabilized with different stabilizers. Different uppercase letters indicate significant differences in creaming index at day 1. Different lowercase letters indicate significant differences in creaming index at day 7. (*p* < 0.05 by Duncan’s multiple range test). CMC = sodium carboxymethyl cellulose, BSG = brewers’ spent grain.

**Figure 4:**
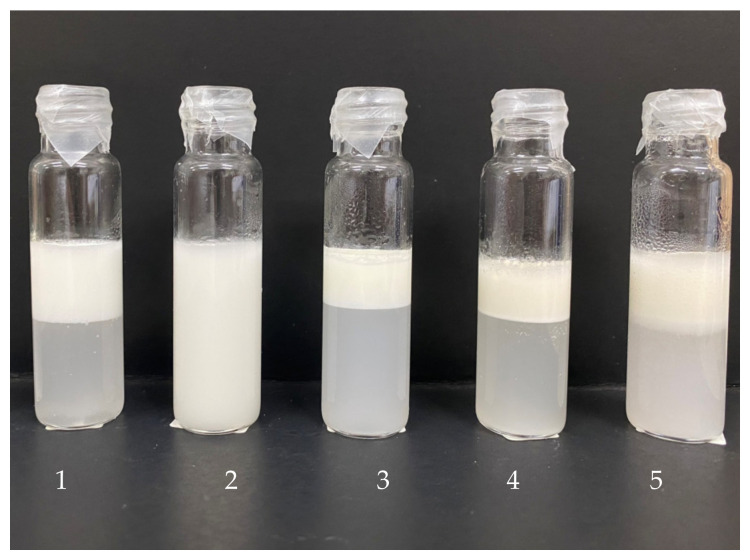
Visual appearance of emulsions stabilized with different stabilizers at a concentration of 0.4% (*w*/*v*) after 7 days of storage at room temperature. 1—CMC, 2—Xanthan gum, 3—Gum arabic, 4—Oat β-glucan, 5—BSG β-glucan. CMC = sodium carboxymethyl cellulose, BSG = brewers’ spent grain.

The creaming index and the visual appearance of the emulsions stabilized with different stabilizers after 7 days of storage are shown in Figure 3 and Figure 4, respectively. The emulsion stabilized with xanthan gum exhibited the highest stability, as shown by the lowest creaming index. To some extent, the emulsions stabilized with oat and BSG β-glucans were stable. Emulsion stability is also affected by the concentration of the stabilizer and its protein impurities. The extracted BSG β-glucan contained 5.37% protein as determined by the Bradford assay [25]. According to Karp et al. [23], the high stability of emulsions stabilized by cereal β-glucan depends on the properties of the polysaccharides. High molecular weight polysaccharides can thicken, form a gel-like polymer network, and stabilize emulsions. Barley-β-glucan prevents flocculation and aggregation of emulsions and also improves emulsion stability by increasing emulsion viscosity [26].

**Figure 5:**
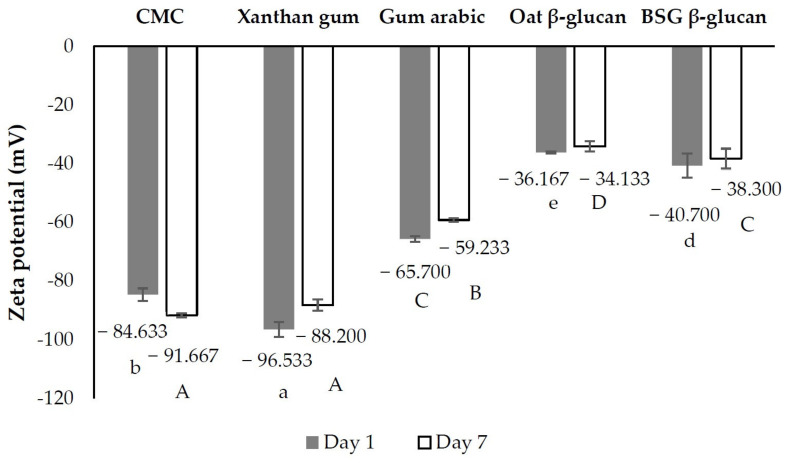
Zeta potential of emulsions stabilized with different stabilizers. Different lowercase letters indicate significant differences in creaming index at day 1. Different uppercase letters indicate significant differences in creaming index at day 7. (*p* < 0.05 by Duncan’s multiple range test). CMC = sodium carboxymethyl cellulose, BSG = brewers’ spent grain.

The zeta potential of the emulsions stabilized with different stabilizers is shown in Figure 5. Zeta potential measurements with absolute values of less than 30 mV and more than 30 mV indicate flocculated and deflocculated emulsions, respectively [27]. The measured absolute values were all greater than 30 mV, with the emulsion stabilized with xanthan gum having the highest value, resulting in the highest stability of the emulsion after 7 days of storage (Figure 3 and Figure 4). The CMC stabilized emulsion had the second highest zeta potential and the second highest emulsion stability. The other emulsions with lower absolute zeta potential values showed significantly lower emulsion stability. It is generally known that the zeta potential value correlates with the creaming index [28]. In addition, the viscosity of the continuous phase was increased by the addition of various stabilizers, which led to a reduction in the kinetic motion of the oil droplets as well as the rate of flocculation and coalescence and an increase in emulsion stability (Figure 3). This observation suggests the presence of a gel-like property within the polymer, influencing emulsion stability in addition to the zeta potential.

Zeta potential and viscosity could exert a synergistic effect on emulsion stability, and a linear correlation may not be suitable for establishing the relationship between emulsion stability and these parameters. A non-linear relationship was demonstrated in the work of Genovese and Lozano [29]. To primarily assess the relationship between these parameters, a Pearson correlation was conducted to evaluate the linear correlation between the pairs of data, which are illustrated in Table 2. However, certain sets of data exhibited a non-linear relation. For example, viscosity appeared to have an exponential relationship with water holding capacity and creaming index. After conducting Pearson correlation analysis to evaluate the relationships among viscosity in water, creaming index at day 1, and zeta potential at day 1 upon the addition of these stabilizers in the emulsion, no significant relationships were observed between any pair of variables (*p* > 0.050).

There are no significant relationships between any pair of variables in the correlation table (*p* > 0.050).

### 3.3. Application of BSG β-Glucan in Orange Juice Beverage

The application of BSG β-glucan as a stabilizer in a prototype orange juice beverage was evaluated and compared with other commercial stabilizers. The beverages were examined for their zeta potential, color, and viscosity.

#### 3.3.1. Viscosity

**Figure 6:**
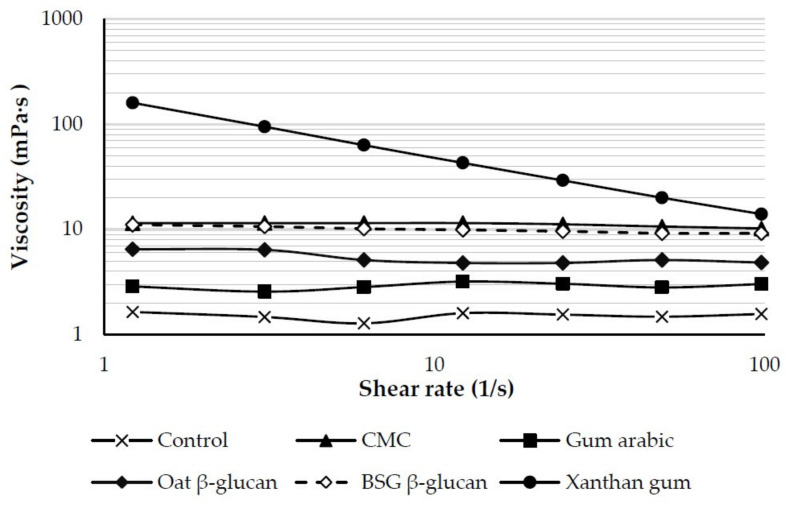
Apparent viscosity as a function of shear rate of orange juice without (control) and with various stabilizers. CMC = sodium carboxymethyl cellulose, BSG = brewers’ spent grain.

The results of apparent viscosity are shown in Figure 6. The apparent viscosity of orange juice is affected by the type and concentration of the stabilizer, which should be selected appropriately for beverage products due to the differing molecular weights of each stabilizer [30]. The viscosity of the orange juice increased with the addition of various stabilizers in the following order: xanthan gum > CMC > BSG β-glucan > oat β-glucan > gum arabic > control. All orange juice samples without (control) and with various stabilizers, with the exception of xanthan gum, showed nearly constant viscosity with increasing shear rate, demonstrating Newtonian flow behavior. However, the sample stabilized with xanthan gum exhibited pseudoplastic behavior. Similarly, Mirhosseini et al. [31] reported that the high apparent viscosity of an orange beverage emulsion was caused by the high concentration of xanthan gum. The complicated structure of xanthan gum, which has a high molecular weight and many free carboxyl groups, leads to a high water absorption capacity and shows pseudoplastic behavior at high shear rates. Hydrocolloids used as stabilizers in beverages can increase viscosity and form a gel that retards the movement of the pulp or immobilizes it, resulting in increased stability of the beverages [32].

#### 3.3.2. Color

**Figure 7:**
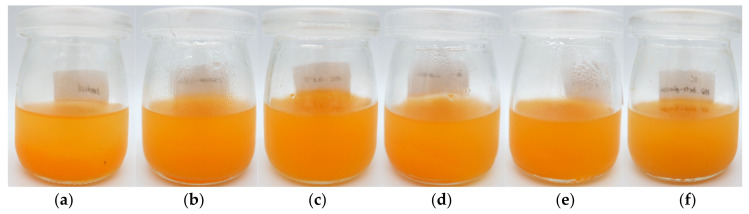
Precipitation and suspension of orange pulp in orange juice without (control) and with various added stabilizers. From left to right: (**a**) control, (**b**) xanthan gum, (**c**) CMC, (**d**) gum arabic, (**e**) oat β-glucan, and (**f**) BSG β-glucan. CMC = sodium carboxymethyl cellulose, BSG = brewers’ spent grain.

The orange juice stabilized with various stabilizers had higher lightness (L*), redness (a*), and yellowness (b*) than the control sample (Table 3). The control sample had a higher precipitation of orange pulp than the samples containing stabilizers or thickeners (Figure 7). The orange juice stabilized with BSG β-glucan had the highest lightness, redness, and yellowness.

To investigate how these stabilizers alter the color of the prototypes, the color parameters of the various stabilizers dissolved in water were measured and these are shown in Table 4. The BSG β-glucan solution showed the highest opacity (highest L* value) and color (highest a* and b* values). Therefore, the color of the orange juice was mainly influenced by the color of the stabilizer itself. Since the BSG β-glucan is opaque, it gives a lighter product than the other stabilizers.

According to Temelli et al. [33], the addition of higher barley β-glucan concentrations to orange juice beverages resulted in higher lightness values of the product due to turbidity. Therefore, β-glucan can be used as a clouding agent in functional beverage products.

#### 3.3.3. Zeta Potential of Beverage

**Figure 8:**
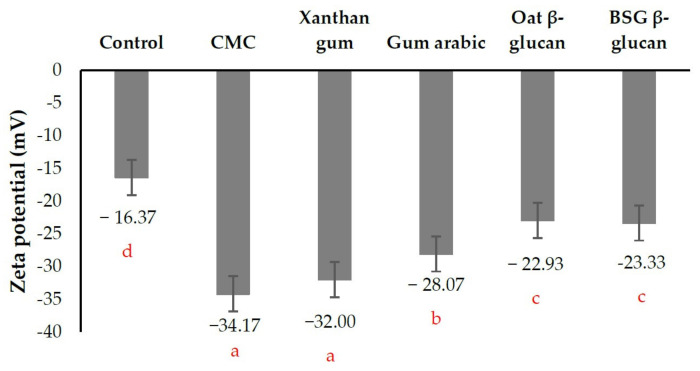
The zeta potential of the prototype orange juice beverage without (control) and with different stabilizers. Different letters indicate significant differences (*p* < 0.05 by Duncan’s multiple range test). CMC = sodium carboxymethyl cellulose, BSG = brewers’ spent grain.

The zeta potential is the value that indicates the surface potential of a droplet. The zeta potential of orange juice without (control) and with different stabilizers is shown in Figure 8. Orange juice stabilized with CMC and xanthan gum had the highest absolute zeta potential values, resulting in the lowest precipitation of orange pulp (Figure 7). It is obvious that the homogeneity of orange juice is the highest. This can be explained by the fact that xanthan gum, a negatively charged polysaccharide, increases the electrostatic and steric forces that cause the droplets to repel each other and prevent the instability of the orange juice [34]. The control sample with the lowest absolute zeta potential value showed extensive precipitation of orange pulp (Figure 7). The orange juice containing β-glucans from oats and BSG had similar absolute zeta potential values that were higher than those of the control, resulting in higher stability. Moreover, these β-glucans increased viscosity and/or formed a gel that facilitated the suspension of the orange pulp in the orange juice. These findings are in good agreement as the previous section.

## 4. Conclusions

The influence of temperature and time on BSG β-glucan extraction is examined in this study. Temperature and time were both found to affect the yield and purity of the extracted β-glucan. At 60 °C and an extraction time of 90 min, the highest yield and purity were obtained. The characteristics of BSG β-glucan were compared with those of oat β-glucan, carboxymethyl cellulose (CMC), gum arabic, and xanthan gum. All samples were examined for viscosity, water holding capacity (WHC), and emulsion stabilizing capacity. The results showed that all aqueous solutions exhibited non-Newtonian behavior over the shear rate range studied (1.22–97.8 s^−1^), with the exception of gum arabic, which exhibited Newtonian behavior. The viscosity, WHC, and emulsion stabilizing capacity were found to be in the same order as follows: xanthan gum > CMC > BSG β-glucan > oat β-glucan > gum arabic. These stabilizers were also evaluated for their ability to stabilize orange juice beverages. All stabilizers improved the color and increased the stability of orange juice by reducing the precipitation of orange pulp. The orange juice stabilized with BSG β-glucan exhibited the highest lightness and color values. Thus, the BSG β-glucan could be suitable for cloudy beverages. As far as we know, this study is the first to report on the potential applications of BSG β-glucan as a functional ingredient in the real food system compared to commercially available stabilizers.

## Figures and Tables

**Table 1:** Effect of temperature and time on β-glucan extraction and yield of product.

Condition		Yield of the Extract (%, *w*/*w*)	β-Glucan Content of the Extract (%, *w*/*w*)	Extraction Yield of β-Glucan (%, *w*/*w*)	Recovery Yield of β-Glucan (%, *w*/*w*)
Temperature (°C)	Time (min)				
45	30	0.087 ± 0.004 ^fgh^	48.305 ± 9.524 ^bcd^	0.042 ± 0.010 ^abc^	9.490 ± 2.305 ^abc^
	60	0.088 ± 0.045 ^cd^	55.419 ± 3.123 ^ab^	0.050 ± 0.027 ^abc^	11.160 ± 6.166 ^abc^
	90	0.135 ± 0.008 ^ab^	42.203 ± 1.628 ^def^	0.057 ± 0.001 ^ab^	12.830 ± 0.269 ^ab^
	120	0.148 ± 0.004 ^a^	34.658 ± 0.221 ^f^	0.051 ± 0.001 ^abc^	11.500 ± 0.212 ^abc^
60	30	0.085 ± 0.002 ^fgh^	37.744 ± 0.153 ^ef^	0.032 ± 0.001 ^c^	7.220 ± 0.141 ^c^
	60	0.081 ± 0.012 ^gh^	39.331 ± 1.964 ^ef^	0.032 ± 0.006 ^c^	7.200 ± 1.428 ^c^
	90	0.096 ± 0.001 ^defgh^	59.837 ± 0.054 ^a^	0.057 ± 0.001 ^a^	12.855 ± 0.177 ^a^
	120	0.090 ± 0.015 ^efgh^	53.359 ± 4.248 ^abc^	0.048 ± 0.012 ^abc^	10.830 ± 2.673 ^abc^
75	30	0.134 ± 0.002 ^ab^	41.664 ± 2.918 ^def^	0.056 ± 0.005 ^abc^	12.535 ± 1.068 ^abc^
	60	0.079 ± 0.012 ^h^	41.536 ± 3.644 ^def^	0.033 ± 0.008 ^bc^	7.435 ± 1.789 ^bc^
	90	0.099 ± 0.011 ^defg^	44.941 ± 0.764 ^def^	0.045 ± 0.006 ^abc^	10.020 ± 1.315 ^abc^
	120	0.128 ± 0.003 ^bc^	41.254 ± 2.376 ^def^	0.053 ± 0.002 ^abc^	11.845 ± 0.375 ^abc^
90	30	0.107 ± 0.031 ^ab^	45.807 ± 3.391 ^cde^	0.050 ± 0.018 ^abc^	11.165 ± 3.953 ^abc^
	60	0.084 ± 0.006 ^fgh^	48.319 ± 0.897 ^bcd^	0.041 ± 0.002 ^abc^	9.090 ± 0.509 ^abc^
	90	0.107 ± 0.003 ^de^	38.500 ± 5.640 ^ef^	0.041 ± 0.007 ^abc^	9.270 ± 1.626 ^abc^
	120	0.103 ± 0.001 ^def^	45.706 ± 1.590 ^cde^	0.047 ± 0.002 ^acb^	10.600 ± 0.509 ^abc^

Each value represents the mean ± SD of triplicate experiments. Different superscripts with the same columns are significantly different (*p* < 0.05 by Duncan’s multiple range test).

**Table 2:** Pearson correlation coefficients (r) between viscosity, creaming index at day 1, and zeta potential at day 1.

	Viscosity in Water	Creaming Index	Zeta Potential
Viscosity in water	1		
Creaming index	−0.818	1	
Zeta potential	0.79	−0.855	1

**Table 3:** Color parameters of the orange juice that contained different stabilizers.

Sample	L*	a*	b*
Control	52.82 ± 0.78 ^d^	15.54 ± 0.42 ^d^	51.59 ± 0.60 ^c^
Xanthan gum	60.09 ± 0.46 ^c^	16.60 ± 0.05 ^bc^	53.72 ± 1.41 ^bc^
CMC	67.31 ± 1.17 ^b^	16.55 ± 0.08 ^bc^	52.64 ± 1.58 ^bc^
Gum arabic	61.82 ± 0.78 ^c^	16.13 ± 0.10 ^c^	48.93 ± 1.01 ^d^
Oat β-glucan	60.08 ± 0.79 ^c^	16.88 ± 0.01 ^ab^	55.13 ± 0.95 ^b^
BSG β-glucan	70.09 ± 0.55 ^a^	17.18 ± 0.11 ^a^	58.71 ± 0.04 ^a^

L* = the lightness of the product with L* = 100 as white and L* = 0 as black; a* = red (+) or green (*−*) direction; b* = yellow (+) or blue (*−*) direction. Different superscripts with the same columns are significantly different (*p* < 0.05 by Duncan’s multiple range test). CMC = sodium carboxymethyl cellulose, BSG = brewers’ spent grain.

**Table 4:** Color parameters of stabilizers or thickeners dissolved in water at a concentration of 0.5% (*w*/*v*).

Sample	L*	a*	b*
Xanthan gum	60.09 ± 0.46 ^b^	0.27 ± 0.09 ^b^	1.99 ± 0.11 ^b^
CMC	61.31 ± 1.17 ^b^	0.05 ± 0.02 ^c^	0.85 ± 0.07 ^d^
Gum arabic	61.80 ± 0.76 ^b^	0.12 ± 0.03 ^bc^	0.98 ± 0.04 ^d^
Oat β-glucan	57.08 ± 0.79 ^c^	0.32 ± 0.12 ^b^	1.27 ± 0.07 ^c^
BSG β-glucan	65.94 ± 0.34 ^a^	0.72 ± 0.06 ^a^	3.23 ± 0.18 ^a^

L* = the lightness of the product with L* = 100 as white and L* = 0 as black; a* = red (+) or green (−) direction; b* = yellow (+) or blue (−) direction. Different superscripts with the same columns are significantly different (*p* < 0.05 by Duncan’s multiple range test). CMC = sodium carboxymethyl cellulose, BSG = brewers’ spent grain.

## Data Availability

Data is contained within the article or Supplementary Material.

## Supplementary Materials

The following are available online at https://www.mdpi.com/article/10.3390/foods13030440/s1, Figure S1: The relation between viscosity and creaming index at day 1 fits the exponential decay. Figure S2: The relation between viscosity and WHC appeared to fit exponential rise to maximum $y=y_{0}+a^{*}\left(1-e^{-bx)}\right)$. Figure S3: ImageJ analysis of the prototypes at 0 and 24 h.
