# Supplementary material for: Valorization of Spent Grains from Beer Production through β-Glucan Extraction
Source: Foods. 2024 Jan 29;13(3):440. doi: 10.3390/foods13030440 (PMC10855925; doi:10.3390/foods13030440)
Supplement: Supplementary file 1 [file foods-13-00440-s001.zip › foods-2747122-supplementary.pdf]

1. Sodium Carboxymethyl Cellulose (CMC)

KC กรุงเทพเคมี KRUNGTHEPCHEMI.COM
73 Yu Yen Road Khaewng Ladprao Khet Ladprao, Bangkok 10230
เลขที่ 73 ถนนอยู่เย็น แขวงลาดพร้าว เขตลาดพร้าว กรุงเทพมหานคร 10230
Tel. 02-015-6262, 093-038-3875 E-mail : Sale@krungthepchemi.com
โทร. 02-015-6262, 093-038-3875 อีเมล : Sale@krungthepchemi.com

PRODUCT SPECIFICATION

SODIUM CARBOXYMETHYL CELLULOSE （CMC）

Property	Specification
Physical form	White or cream colored powder
Viscosity (1%solution mpa.s, 25℃)	1800-2000
PH	6.5-8.5
D·S	≥0.90
Purity %	≥99.5
Loss after drying(H2O),%	≤8.0
Sodium	< 12.4%
Sodium Chloride	< 0.5%
Free Glycolate	< 0.4%
Heavy Metal(as Pb),%	≤0.0015
Iron(Fe),%	≤0.02
Arsenic(As),%	≤0.0002
Lead(Pb),%	≤0.0002

2 Xanthan gum

KC กรุงเทพเคมี KRUNGTHEPCHEMI.COM

73 Yu Yen Road Khaewng Ladprao Khet Ladprao, Bangkok 10230
เลขที่ 73 ถนนอยู่เย็น แขวงลาดพร้าว เขตลาดพร้าว กรุงเทพมหานคร 10230
Tel. 02-015-6262, 093-038-3875 E-mail : Sale@krungthepchemi.com
โทร. 02-015-6262, 093-038-3875 อีเมล : Sale@krungthepchemi.com

Specification

Xanthan Gum Food Grade

DESCRIPTION:

Xanthan Gum produced by fermentation of carbohydrate with Xanthomonas campestris, its solutions are neutral, suitable for use in food and food preparations as stabilizer, thickener emulsifier.

SPECIFICATION:

Properties	Specification
Appearance	cream colored powder
Assay (%)	91.0% - 117%
Viscosity (1% solution in 1% KCL)	1200-1600cps
PH (1% solution)	6.0-8.0
Solubility	soluble in waer: insoluble in ethanol
Gel formation	pass test
Loss on Drying(%)	max. 15%
Ash	6.5-16%
Particle size	100% through 60 mesh(250micro)<br>min 95% through 80 mesh(180 mocro)
V1/V2	1.02-1.45
Nitrogen (%)	max. 1.5%
Ethanol and Isopropyl Alc.	max. 500ppm
Pyruvic acid	min. 1.5%
Heavy metal	max. 20ppm
Lead	max. 2ppm
Arsenic	max.3ppm
Microbiological	
Total plate count	not more than 2000cfu/g
Yeast/mould	not more than 100cfu/g
E.coli	absent/25g
Salmonella	absent/25[illegible]
Xanthomonas campestris	Viable cells absent in 1g

*We hereby confirm that our product is certified that meet the standard requirement of Codex advisory specification for the identity and purity of Food Additives

PACKAGE:

Net 25Kg carton box, multiply paper bag or cardbord drum with inner blue PE bags

STORE:

Sealed and stored in cool, dry conditions.

SHELF LIFE: Two years

Regulatory Compliance : FCC, E415

KC กรุงเทพเคมี KRUNGTHEPCHEMI.COM

3 Gum Arabic

KC กรุงเทพเคมี KRUNGTHEPCHEMI.COM

73 Yu Yen Road Khaewng Ladprao Khet Ladprao, Bangkok 10230
เลขที่ 73 ถนนอยู่เย็น แขวงลาดพร้าว เขตลาดพร้าว กรุงเทพมหานคร 10230
Tel. 02-015-6262, 093-038-3875 E-mail : Sale@krungthepchemi.com
โทร. 02-015-6262, 093-038-3875 อีเมล : Sale@krungthepchemi.com

SPECIFICATION CERTIFICATE

PRODUCT NAME	:	GUM ARABIC<br>HS. CODE : 1301.20.00
SYNONYMS	:	GUM ARABIC, GUM HASHAB, ACACIA SENEGAL KORDOFAN, INS 414, C.A.S. NO.9000-01-5
QUALITY	:	100% GUM ARABIC (ACACIA SENEGAL), PRODUCED BY SIEVING AND CRUSHING INTO UNIFORM SIZE RANGE FROM ABOUT < 120 MICRO. TAILOR MADE ACCORDING TO THAT REQUIREMENT. PHYSICAL AND PROPERTIES ARE THAT RAW MATERIAL EXCEPT THAT PRODUCT IS VERY CLEAN FROM PARK, SAND OR FOREIGN MATERIALS.
PHYSICAL	:	DRIED GUMMY EXUDATION ODOURLESS FROM THE STEM AND DOES NOT AFFECT THE FLAVOR, ODOUR OR COLOR OF THE SOLUTION.
COMPOSITION	:	COMPLEX POLYSACCHARIDE, ON HYDROLYSIS YIELDS. GALACTOSE, RHAMNOSE, ARABINOSE AND GLUCURONIC WITH AROUND 2% OF PROTEIN AND TRACE OF SODIUM, MAGNESIUM. ACID CALCIUM AND POTASSIUM SALT.
APPEARANCE	:	WHITE OR YELLOWISH-WHITE POWDER
FUNCTIONAL USES	:	EMULSIFIER, STABILIZER, THICKENER
CHARACTERISTICS	:	HAND-PICKED AND HAND SELECTED, PRE-CLEANED BY HAMMERING AND SCREENING TO THE SIZE. HIGH QUALITY MEETING ALL FOODS, PHARMACEUTICAL AND COSMETIC.
SOLUBILITY (VOL.4)	:	SOLUABLE IN WATER, INSOLUBLE IN ETHANOL
LOSS ON DRYING (VOL.4)	:	NOT MORE THAN 10%
TOTAL ASH (VOL.4)	:	NOT MORE THAN 4%
ACID-INSOLUBLE ASH	:	NOT MORE THAN 0.5%
ACID-INSOLUBLE MATTER	:	NOT MORE THAN 1%
PH (%)	:	4.0 - 4.5
VICOSITY (cps)	:	60 - 110
ARSENIC	:	NOT MORE THAN 3 mg/kb
LEAD (Pb)	:	NOT MORE THAN 2 mg/kg
SALMONELLA SPP.	:	NEGATIVE
E.COLI	:	NEGATIVE
LEAD	:	NOT MORE THAN 2mg/kg
PACKING	:	25 KGS. PACKED IN 4-POLY PAPER BAGS WITH PE LINER. PACKED IN EXPORT CARTON SIZE 16"X18.5"X9" . LOADED WITH OR WITHOUT PALLETS.
SHELF LIFE	:	6 YEARS

4. Oat β-glucan

REBECCA

Shaanxi Rebecca Bio-tech Co., Ltd

www.rebeccabio.com Fax: +86-029-85219166 Email: sales001@sxrebecca.com
Rm.2810, Lanshan Gongguan, No.3, Nonglin Rd, ChangAn South Rd, Yanta District, Xi`An, Shaanxi, P.R. China.

CERTIFICATE OF ANALYSIS

**Product Name**	Oat Beta Glucan
**Botanical source**	*Avena Sativa*
**Plant part used**	Seed
**Batch Number**	REB210520
**Quantity**	500KG
**Production Date**	May.20,2021
**Report Date**	May.21,2021
**Expiration Date**	May.19,2023

ANALYSIS	SPECIFICATION	RESULTS
Appearance	White to light yellow powder	Complies
Odor	Slight characteristic odor or odorless	Complies
Sieve analysis	NLT 95% pass 80 mesh	Complies
Oat β-Glucan	≥70%	71.3%
Protein	≤8%	5.68%
Fattiness	≤1%	0.096%
Loss on drying	≤5%	3.4%
Heavy metals		
Pb	≤1ppm	Complies
As	≤1ppm	Complies
Cd	≤0.2ppm	Complies
Hg	≤0.2ppm	Complies
Microbiology		
Aerobic plate count	≤1000cfu/g	＜400cfu/g
Yeast & Mould	≤150 cfu/g	＜100cfu/g
Thermotolerant coliforms	Negative	Negative
Salmonella	Negative	Negative
Staphylococcus aureus	Negative	Negative
**Conclusion**	**Conform with specification.**	
**Packing**	Packed in paper-drums (N.W.: 25KG) and two plastic-bags inside.	
**Storage**	Keep in cool & dry place. Keep away from strong light and heat.	
**Shelf life**	24 months under the conditions above and in its original packaging.	

Analyst : FENG XIAONI Checker : LI QIANG Approved: HU JIANMIN

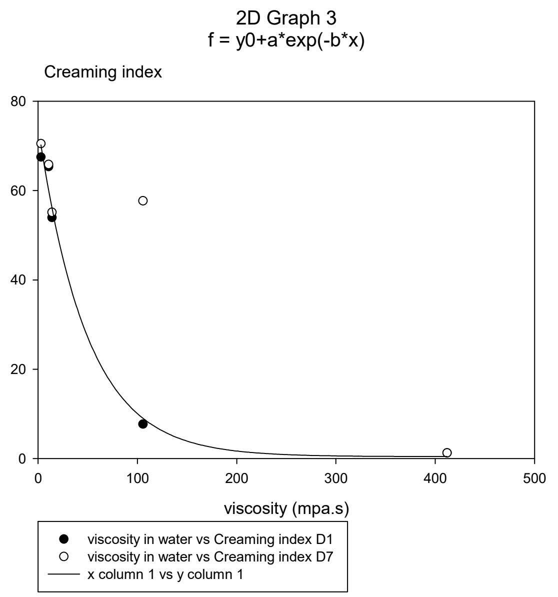

Figure S1. The relation between viscosity and creaming index at day 1 fits the exponential decay.

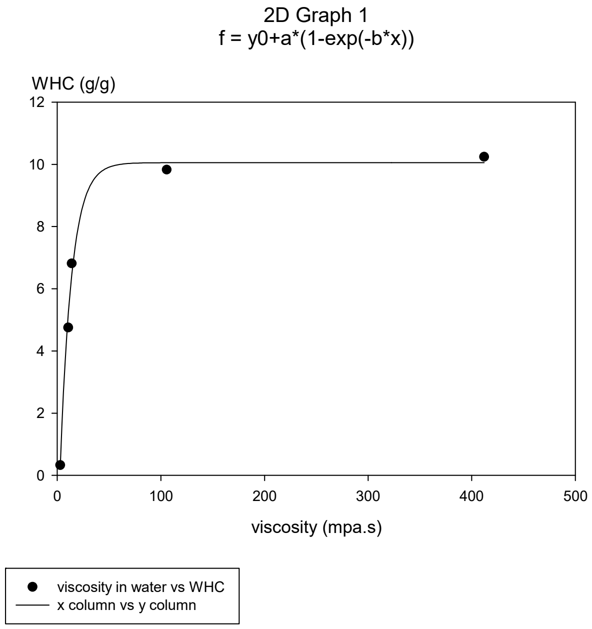

Figure S2. The relation between viscosity and WHC appeared to fit exponential rise to maximum $y = y_0 + a^*(1 - e^{-bx})$

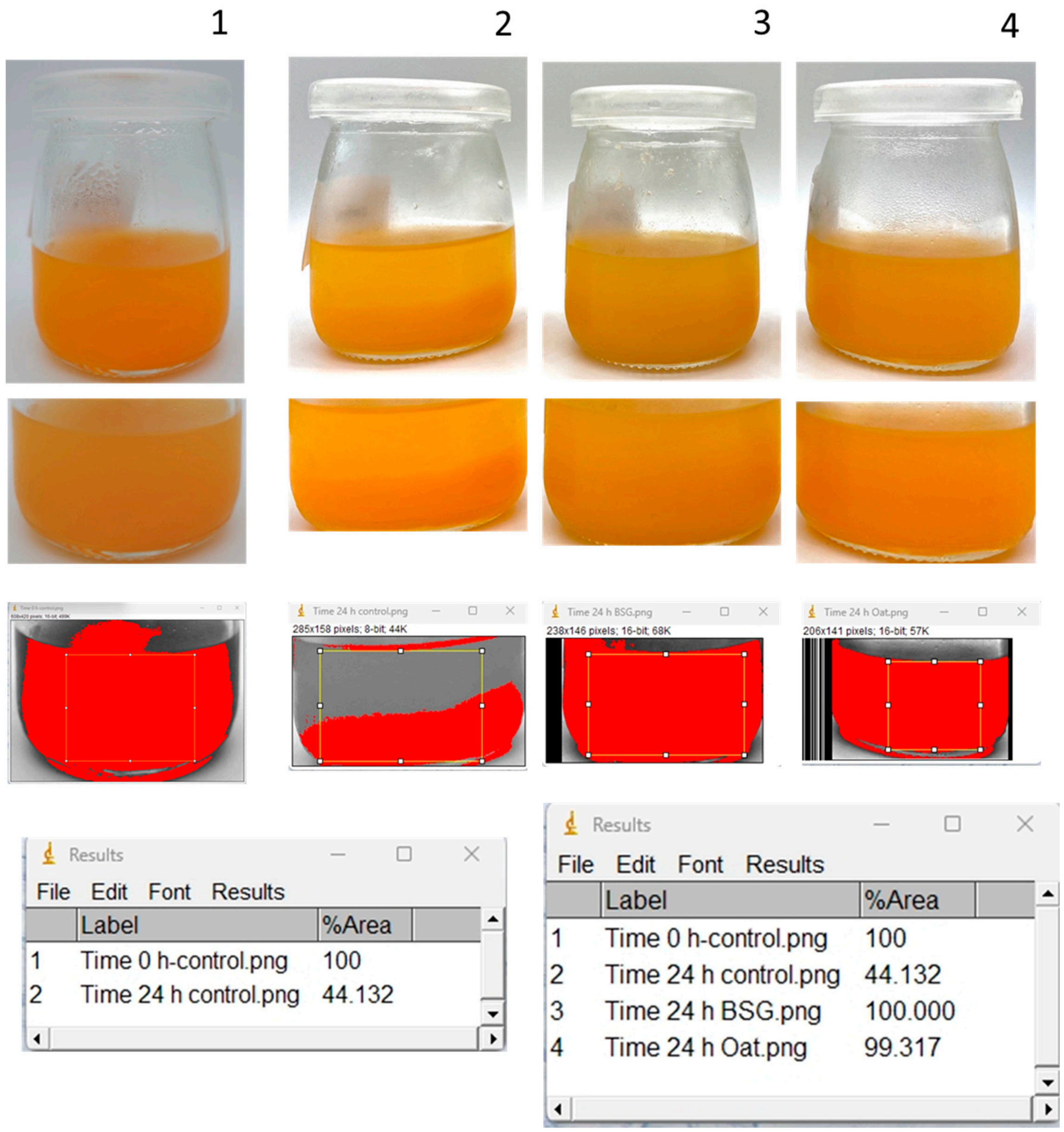

Figure S3: ImageJ analysis of the prototypes at 0 and 24 hours.

Area fraction of selected area

1. Time 0: control
2. Time 24: control
3. Time 24: BSG
4. Time 24: Oat beta-glucan

The threshold was set at 0-120. The selected areas are in the rectangular.
